# Virus-Specific Secondary Plasma Cells Produce Elevated Levels of High-Avidity Antibodies but Are Functionally Short Lived

**DOI:** 10.3389/fimmu.2019.01831

**Published:** 2019-08-06

**Authors:** Caroline C. Krueger, Franziska Thoms, Elsbeth Keller, Monique Vogel, Martin F. Bachmann

**Affiliations:** ^1^Department of Rheumatology, Immunology and Allergology, Inselspital, Bern University Hospital, University of Bern, Bern, Switzerland; ^2^Department for BioMedical Research, University of Bern, Bern, Switzerland; ^3^Department of Dermatology, University Hospital Zurich, Schlieren, Switzerland; ^4^Nuffield Department of Medicine, The Jenner Institute, The Henry Wellcome Building for Molecular Physiology, University of Oxford, Oxford, United Kingdom

**Keywords:** adaptive immunity, anti-viral immunity, memory B cells, secondary plasma cells, virus-like particles

## Abstract

Most vaccines aim at inducing durable antibody responses and are designed to elicit strong B cell activation and plasma cell (PC) formation. Here we report characteristics of a recently described secondary PC population that rapidly originates from memory B cells (MBCs) upon challenge with virus-like particles (VLPs). Upon secondary antigen challenge, all VLP-specific MBCs proliferated and terminally differentiated to secondary PCs or died, as they could not undergo multiple rounds of re-stimulation. Secondary PCs lived in bone marrow and secondary lymphoid organs and exhibited increased production of antibodies with much higher avidity compared to primary PCs, supplying a swift wave of high avidity antibodies early after antigen recall. Unexpectedly, however, secondary PCs were functionally short-lived and most of them could not be retrieved in lymphoid organs and ceased to produce antibodies. Nevertheless, secondary PCs are an early source of high avidity antibodies and induction of long-lived MBCs with the capacity to rapidly differentiate to secondary PCs may therefore be an underestimated possibility to induce durable protection by vaccination.

## Introduction

B cells differentiate to antibody secreting plasma cells (PCs) upon activation by their cognate antigen (Ag) within and outside of B cell follicles. At an early stage of the primary immune response, antibody–forming cells (AFCs) derived from follicular or marginal zone (MZ) B cells are rather short-lived and survive for a few days only (1). Meanwhile, follicular B cells form GCs where MBCs and long-lived PCs are generated in a mostly T cell dependent fashion (2–5).

Activated B-lymphocytes are driven to the PC pathway by up-regulation of the transcription factors B lymphocyte maturation protein 1 (Blimp-1), Interferon regulating protein 4 (IRF 4), and X-box-binding protein 1 (XBP 1) (6–8). Differentiation of activated B cells into AFCs needs a harmonized change in the gene expression of these cells. Shi et al. delineated the transcriptional profile during this differentiation process (9). PCs are terminally differentiated and arrested in the G1 phase of the cell cycle being incapable of further growth or proliferation (10, 11). To be able to secrete large amounts of antibodies, PCs are committed to their protein synthesizing machinery and undergo major structural adaptations by increasing the size of the endoplasmic reticulum and Golgi apparatus (12). To cope with these changing conditions PCs induce the unfolded protein response as well as autophagy (13–15). These stress-regulating processes are necessary for survival as PCs can secrete the tremendous amount of up to 10'000 antibodies per second (16). Sizeable amounts of antibodies that are rapidly available are required to neutralize microorganisms and prevent infection. Antibodies furthermore play a key role in immunity and promote the crosstalk between the innate and adaptive immune system. Besides classical neutralization of toxins and pathogens, they are able to opsonize microbes and infected cells for phagocytosis, enabling their elimination, and promote antigen presentation thereby regulating inflammation (17).

PCs are found in secondary lymphoid organs and the bone marrow (BM) where they can survive for days, months, or even years. There is an ongoing debate whether long-term antibody responses are a result of persisting antigen leading to re-stimulation and differentiation of memory B cells to PCs or whether they are derived from intrinsically long-lived PCs. Several studies are in favor of the first hypothesis that persistent antigen or infection and polyclonal memory B cell activation is required (18–22). Nevertheless, evidence is growing that PCs can persist in the absence of continuous stimulation (23–25). It was shown that PCs require cell-intrinsic and extrinsic survival signals such as cytokines and adhesion molecules from nursery cells like monocytes, eosinophils, and megakaryocytes for long-term survival in BM niches (26–29). Once they reach the BM and successfully compete for a niche, PCs have a lifespan varying from a few months to years and even decades during which they constantly secrete antibodies (30, 31). In contrast to PCs, which do not express surface Ig, MBCs respond to secondary Ag encounter. They exhibit the intrinsic ability to respond with a proliferative burst faster compared to naïve B cells (32) and were found to seed new GCs and/or differentiate into PCs (33–37). Antibody responses generated during secondary responses are usually of higher affinity for the cognate Ag compared to those of a primary response.

We have previously shown that immunization with VLPs derived from the RNA bacteriophage Qβ elicit strong and sustained IgG antibody responses by activation of MZ and follicular B cells with the latter forming GCs (38–40). MBCs and PCs were rapidly generated and detectable as early as 3 days and up to several months after immunization in spleen and BM (41, 42). Here we show, that MBCs generated against Qβ proliferated during Ag recall experiments but exclusively differentiated into secondary PCs and failed to respond to multiple rounds of Ag stimulation. Secondary PCs exhibited the unique ability to produce 30 times more antibodies of increased affinity compared to primary PCs. The secondary PCs were found in spleen as well as in BM early on day 4 but almost completely disappeared by day 6 after Ag re-encounter from both organs. In addition, antibodies produced by secondary PCs were cleared from the system within weeks indicating that secondary PCs are functionally short-lived. Inducing MBCs that differentiate into secondary PCs by vaccination could represent a novel pathway for efficient and rapid control of infectious diseases by the induction of an early wave of high affinity antibodies.

## Materials and Methods

### Study Design

The goal of this study was to further characterize secondary PCs, which were generated by MBCs after Ag challenge. To achieve this, adoptive transfers in allotypic mice (Ly5.1/Ly5.2 and IgHa/IgHb) were performed. This enabled us to study primary and secondary immune responses in the same animal. All mice were kept according to Cantonal Veterinary guidelines at the central animal facility (Department for BioMedical Research) of the University of Bern and controlled laboratory experiments were performed in accordance with ethical principles and guidelines of the Cantonal Veterinary Office Bern, Switzerland. Animals were randomly assigned to the different groups. MBCs were generated by VLP immunization of mice. The control naïve mice remained untreated. At the same time, B cells were isolated from memory and naive mice and transferred into recipients. Upon immunization with VLPs, serum samples, spleens, and BM were collected and subjected to ELISA, ELISPOT, and FCM analysis. The investigators who performed the experiments, assessed, analyzed, and quantified the results were not blinded and aware of which group a sample was taken from. Individual groups consisted of 4–5 mice. All experiments were performed in at least 2 independent biological replicates, apart from intracellular FCM analysis of PCs at day 6 after challenge. Data were collected at previously determined time points. All data were included in the analysis.

### Mice

C57BL/6JRccHsd wildtype mice were purchased from Envigo (Horst, The Netherlands). The IgHa (B6.Cg-Gpi1 <a> Thy1 <a> Igh <a> (Stock No. 001317)) mouse strain was purchased from the Jackson Laboratory (USA). We thank Prof. Annette Oxenius for the kind donation of the Ly5.1 (B6.SJL-Ptprc <a> Pepc <b>/BoyJ) mouse strain.

### Antigen

The bacteriophage derived Qβ virus-like particles (VLPs) self-assemble and enclose bacterial RNA during their production in *E. coli*. Due to their particulate and repetitive structure, the VLPs are highly immunogenic. The purification process is described elsewhere (43).

### Immunization

To induce primary immune responses and generate MBC against the VLPs, mice were immunized intravenously (i.v.) with 50 μg Qβ VLPs. To challenge adoptively transferred MBC or naive cells, recipient mice were immunized with 50 μg Qβ VLPs i.v. For intravenous administration the VLPs were formulated in 150 μl sterile PBS.

### Adoptive Transfer

MBCs were generated by immunization of congenic donor mice (Ly5.1 or IgHa). At least 8 weeks after immunization donor mice were sacrificed and spleens isolated in RPMI media containing 2% FCS and antibiotics. A single cell suspension of the spleens was prepared and red blood cells were lysed using ACK buffer (0.15 M ammonium chloride, 0.01 M potassium hydrogen carbonate, pH 7.2–7.4). The splenocytes were PNA^−^ and B220^+^ MACS purified. For PNA negative purification splenocytes were labeled using PNA-biotin (Vector Labs, B-1075) and PNA^+^ cells were depleted by Strepravidin MicroBeads (Milteny Biotec, 130-048-101) according to the manufacturer's protocol. Positive selection using B220 MicroBeads (Milteny Biotec, 130-049-501) was performed according to the manufacturer's protocol.

Purified cells from 1/3 of a donor spleen (Ly5.1 or IgHa; ~1–3 × 10^6^ cells) were adoptively transferred i.v. into congenic host mice (Ly5.2 or IgHb). Control mice received PNA^−^ and B220^+^ purified splenocytes from naïve congenic mice. One day after MBC transfer host mice were challenged with 50 μg Qβ VLPs i.v.

### Flow Cytometry (FCM)

For FCM staining tissues (spleen, BM, kidney, lymph nodes (LN), liver, lung) of mice after adoptive transfer were isolated in RPMI supplemented with 2% FCS and antibiotics and single cell suspensions were prepared. Blood was collected in phosphate buffer containing heparin (1–2 units/ml). Red blood cells were lysed using ACK buffer prior to staining. Fc receptors were blocked using an anti-CD16/32 antibody. Qβ specific class switched (CS) B cells were identified as IgM, IgD, CD4, CD8, GR1, CD11b, CD11c negative (all antibodies labeled with phycoerythrin (PE)), and positive for B220 labeled with PE-Cy7 and Qβ VLP labeled with Alexa Flour 488. To discriminate Qβ specific PCs from Qβ specific activated and CS B cells, surface immunoglobulins (Ig) of specific cells were blocked using unlabeled Qβ VLPs. PCs were further stained with and characterized as IgM, IgD, CD4, CD8, GR1, CD11b, CD11c negative (all antibodies labeled with PE) and B220-PE-Cy7 low. To detect Qβ specific PCs by intracellular staining of specific Ig, splenocytes were permeabilized using FACS lysing solution (BD, 349202) containing 0.04% Tween20 and stained with Alexa Flour 488 labeled Qβ VLPs. The congenic marker Ly5.1 (antibody labeled with APC or PerCP-Cy5.5) identified all transfer derived B cells. Dead cells were stained by the addition of propidium iodide solution (PI, Sigma, 10 μg/ml) directly before acquisition. For detection of dead cells after fixation and permeabilisation, the Fixable Viability Dye eFluor 520 (eBioscience, 65-0867-14) was used according to the manufacturer's instructions.

Qβ VLPs were labeled with the Alexa Flour 488 protein labeling kit (Thermo Fisher Scientific, A10235) or Alexa Flour 647 NHS Ester (Thermo Fisher Scientific, A20006) according to the manufacturer's instructions.

Data acquisition was performed on a FACS Canto (BD) and analyzed using FlowJo V10.1 (Flowjo, LLC, USA). All antibodies were purchased from BD Biosciences and Biolegend.

### ELISPOT

Spleens from mice after adoptive transfer were isolated and a single cell suspension was prepared. To collect BM cells, tibia and femur were flushed with RPMI media containing 2% FCS and antibiotics. After red blood cell lysis with ACK buffer, cell numbers of splenocytes and BM cells were determined using the Cellometer mini (Nexcelom, USA). 5 × 10^5^ cells were seeded per well on MAIPS Elispot plates (Millipore, MAIPS4510) previously coated with 10 μg/ml Qβ VLPs overnight at 4°C and blocked with 2% BSA in PBS for at least 2 h. After performing a 2-fold dilution series cells were incubated for 5 h at 37°C and 5% CO_2_. Subsequently cells were washed off and bound specific antibodies produced by PCs were detected using a goat anti-mouse IgG antibody (EY laboratories, AT-2306-2) followed by a donkey anti-goat alkaline phosphatase secondary antibody (Jackson Immunoresearch, 705-055-147). Spots were visualized by the AP Conjugate Substrate Kit (BioRad, 1706432) and counted using an EliSpot Reader (AID, Germany). The spot size was quantified with the EliSpot 7.0 iSpot software of the EliSpot Reader as the average surface area of the spot.

### CFSE Proliferation

To analyse the proliferation of transferred cells, the donor cells were labeled with CFSE (Biolegend, Cat No. 423801) after MACS purification and before transfer into congenic hosts, according to the manufacturer's protocol. FCM staining was carried out similarly as described above. In this case, Qβ specific CS B cells were detected with VLPs labeled with Alexa Flour 647.

### Splenocyte Cell Culture

Spleens from mice that had received memory or naïve B cells were isolated 5 and 6 days after VLP challenge. A single cell suspension of splenocytes was prepared. After red blood cell lysis with ACK buffer, cell numbers of splenocytes were determined using the Cellometer mini (Nexcelom, USA). 10 × 10^6^ cells were seeded in 1 ml RPMI media containing 10% FCS and antibiotics per well in 24 well plates (Falcon Multiwell, Corning). The cells were incubated for 72 h at 37°C and 5% CO_2._ Cell supernatants were harvested and the antibody content determined by ELISA.

### ELISA

Serum samples were obtained from blood collected at the indicated time points during experiments using Microtainer tubes (BD, 365967). Corning half area 96 well-plates were coated with 50 μl of 1 μg/ml Qβ VLPs overnight at 4°C. Sera were 1:10 pre-diluted and 1:4 further serial diluted to analyse a total of 7 dilutions per sample. Qβ specific antibodies were detected using mouse anti-mouse IgG for both allotypes. IgHa-specific (biotin ms anti-ms IgG1[a] (10.9), biotin ms anti-ms IgG2a[a] (8.3) from BD) and IgHb-specific (biotin ms anti-ms IgG1[b] (B68-2), biotin ms anti-ms IgG2a[b] (5.7) from BD) antibodies were detected using horseradish peroxidase (HRP) labeled streptavidin (Dako).

Cell supernatants were used undiluted and a 1:2 serial dilution was performed. An anti-Qβ monoclonal antibody (purified from hybridoma cells) was used as a standard to quantify specific antibodies in the supernatants. Qβ specific antibodies were detected using goat anti-mouse IgG-HRP (Jackson ImmunoResearch, 115-035-071).

The absorbance readings of the tetramethylbenzidine (TMB) color reaction at 450 nm for the serum samples were interpreted as OD50 antibody titers. The OD50 antibody titers are defined as the reciprocal of the dilution that reaches half of the OD max. The anti-Qβ monoclonal antibody standard curve was used to calculate antibody concentrations in the cell supernatants.

### Avidity ELISA

Serum samples were obtained from blood collected at the indicated time points during experiments using Microtainer tubes (BD, 365967). Corning half area 96 well-plates were coated with 50 μl of 1 μg/ml Qβ VLPs overnight at 4°C. Sera of the different time points were applied with a 1:20 pre-dilution and 1:4 further serial diluted. After 1 h incubation, the sera were washed off and the plates washed 3 times 5 min either with 7M urea in PBST (PBS containing 0.05% tween 20) or PBST only. Qβ specific antibodies were detected using mouse anti-mouse IgG for both allotypes. IgHa-specific (biotin ms anti-ms IgG1[a] (10.9), biotin ms anti-ms IgG2a[a] (8.3) from BD) and IgHb-specific (biotin ms anti-ms IgG1[b] (B68-2), biotin ms anti-ms IgG2a[b] (5.7) from BD) antibodies were detected using horseradish peroxidase (HRP) labeled streptavidin (Dako). The absorbance readings of the tetramethylbenzidine (TMB) color reaction at 450 nm served as basis for avidity index calculation. The avidity index (AI) was calculated by AI_x_ = OD (dilution x) + urea / OD (dilution x)–urea.

### Antibodies/Reagents

**Table:** 

**Antibody/reagent**	**Clone**	**Company**	**Conjugate**	**Detection**	**Catalog number**
goat anti-ms IgG	polyclonal	EY laboratories		donkey anti-goat alkaline phosphatase (AP)	AT-2306-2
donkey anti-goat AP	polyclonal	Jackson ImmunoResearch	AP		705-055-147
anti-ms IgG1[a]	10.9	BD	biotin	Streptavidin HRP (Dako)	553500
anti-ms IgG2a[a]	8.3	BD	biotin	Streptavidin HRP (Dako)	553533
anti-ms IgG1[b]	B68-2	BD	biotin	Streptavidin HRP (Dako)	553502
anti-ms IgG2a[b]	5.7	BD	biotin	Streptavidin HRP (Dako)	553504
goat anti-ms IgG	polyclonal	Jackson ImmunoResearch	HRP		115-035-071
anti-ms CD16/32	2.4G2	BD			553142
anti-ms IgM	polyclonal	Jackson ImmunoResearch	PE		115-116-075
anti-ms IgD	11-26c (11-26)	eBioscience	PE		12-5993-83
anti-ms CD8a	53-6.7	BD	PE		553032
anti-ms CD4	H129.19	BD	PE		553653
anti-ms CD11b	M1/70	BD	PE		553311
anti-ms CD11c	HL3	BD	PE		553802
anti-ms GR1	RB6-8C5	BD	PE		553128
anti-ms B220	RA3-6B2	BD	PE-Cy7		552772
anti-ms CD45.1	A20	eBioscience	APC		17-0453-82
anti-ms CD45.1	A20	Biolegend	PerCP/Cy5.5		110727
anti-ms CD38	90	Biolegend	PerCP/Cy5.5		102722
Anti-ms IgG	Polyclonal	eBioscience	Biotin	Streptavidin APC/Cy7	13-4013-85
Peanut Agglutinin (PNA)		Vector Laboratories	Biotin	Streptavidin APC/Cy7	B-1075
Streptavidin HRP		Dako	HRP		P0397
Streptavidin APC/Cy7		BD	APC/Cy7		554063
Fixable Viability Dye eFluor 520		eBioscience	eFluor 520		65-0867-14

### Statistics

Statistical analysis was performed using GraphPad Prism Version 7.01 (GraphPad Software, USA). Statistically significant differences between two groups were calculated using unpaired *t*-tests. Statistically significant differences between more than 2 groups were determined using a one-way ANOVA followed by Tukey's or Sidak's multiple comparisons test. Statistical significance was defined as *p* < 0.05. The best fitting line was calculated by linear regression.

## Results

### Memory B Cell Derived Secondary PCs Produce Antibodies of Higher Avidity

**Figure 1:**
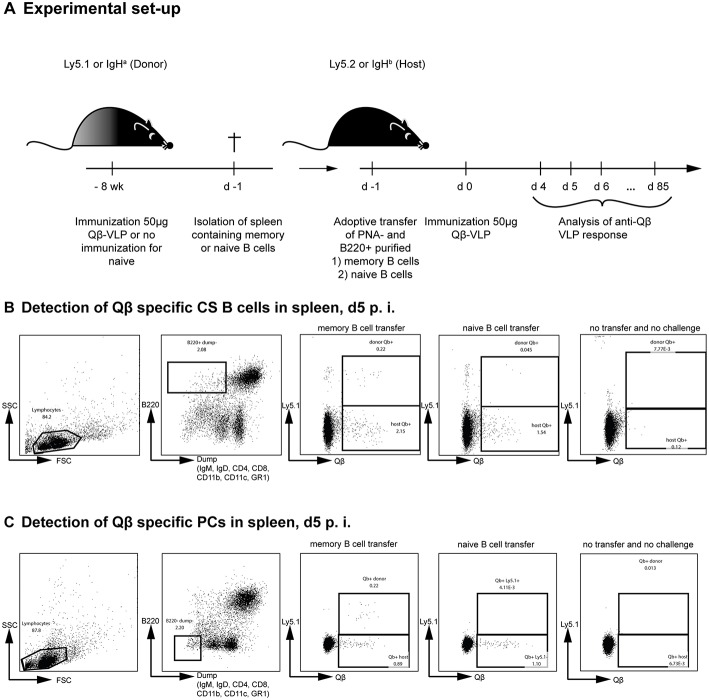
Adoptive transfer of Qβ VLP specific or naïve B cells and flow cytometric analysis of Qβ specific CS B and plasma cells in the spleen. **(A)** Congenic mice (Ly5.1 or IgHa) were immunized with 50 μg Qβ VLPs i.v. Eight weeks after immunization spleens of immunized and naïve mice were isolated and PNA^−^ B220^+^ MACS purified cells were transferred into host mice (Ly5.2 or IgHb). Recipient mice were immunized with 50 μg Qβ VLPs i.v. 1 day after the transfer. Spleens, bone marrow, and serum were taken at several time points after challenge. **(B)** Representative FCM plots for the gating strategy to identify Qβ specific CS B cells in the spleen 5 days after immunization. B220^+^ cells not expressing IgM, IgD, CD4, CD8, CD11b, CD11c, or GR1 were analyzed for their binding of labeled Qβ VLPs. The congenic Ly5 marker was used to discriminate transfer from host derived CS B cells. **(C)** Representative FCM plots for the gating strategy to identify Qβ specific PCs in the spleen 5 days after immunization. B220^low^ cells not expressing IgM, IgD, CD4, CD8, CD11b, CD11c, or GR1 were analyzed for their intracellular binding of labeled Qβ VLPs. The congenic Ly5 marker was used to discriminate transfer from host derived PCs cells.

We have previously shown that MBCs are generated against Qβ VLPs in a T cell-dependent manner (35, 38, 39, 44, 45). During secondary responses, these MBCs do neither extensively proliferate nor join GC reactions (35). T cell help, however, is essential for low-level MBC proliferation but dispensable for differentiation to secondary PCs during secondary immune responses (44). To reveal insights in the mechanism and kinetics of secondary PC formation from MBCs after antigenic re-stimulation, adoptive transfer experiments using congenic mice were performed (Figure 1A). To this end, MBCs were generated by immunizing donor mice (Ly5.1 or IgHa) with 50 μg Qβ VLPs. Eight weeks post immunization, splenocytes from donor mice were isolated and PNA^−^ and B220^+^ MBCs were purified by MACS, excluding transfer of GC B cells. Splenocytes from naïve mice were subjected to the same treatment and served as controls. We have previously shown that the presence of memory T follicular helper cells does not influence the MBC response (35, 44). Therefore, purified MBCs were transferred alone. Donor-derived (Ly5.1^+^) MBCs were shown to preferentially home to secondary lymphoid organs, namely lymph nodes (LN), and spleen (Figure S1A) and the majority of Qβ-specific donor MBCs were found in the spleen (Figure S1B).

**Figure 2:**
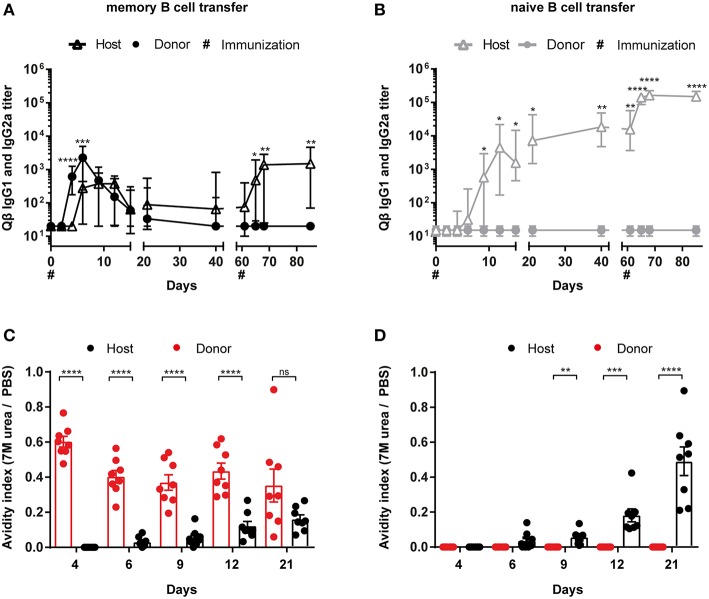
Memory B cell derived secondary PCs produce antibodies of higher avidity. **(A)** MBC responses were initiated by vaccinating IgHa mice with 50 μg Qβ VLPs. After 8 weeks, PNA^−^ and B220^+^ MACS purified B cells from memory **(A)** or naïve **(B)** donor mice were transferred into congenic recipients (IgHb). Recipient mice were challenged with 50 μg Qβ VLPs 24 h and 61 days after the transfer. The anti-Qβ IgG1 and IgG2a antibody titers in the serum were determined by ELISA on days 0, 2, 4, 6, 9, 12, 15, 21, 42, 61, 65, 68, and 85. Using Ha and Hb allotype specific detection antibodies, donor (IgHa), and host (IgHb) responses were discriminated. To determine the avidity index of IgGs in the sera after memory **(C)** or naïve **(D)** B cell transfer and VLP challenge, a modified ELISA was performed. Mean with SEM. *P* values were calculated using an unpaired *t* test. *n* = 4 mice per group. Data representative of 2 independent experiments. *p < 0.05, **p < 0.01, ***p < 0.001, ****p < 0.0001.

To analyse the humoral immune response after memory or naïve B cell transfer and Qβ VLP challenge, immunoglobulin heavy chain allotype mice were used as shown in Figure 1A. MBCs were induced in donor mice (IgHa) and adoptively transferred into recipient mice (IgHb). The recipient mice were challenged with Qβ VLPs 1 day after the transfer and splenocytes, BM as well as serum were collected at the indicated time points to determine CS B cells (outlined in Figure 1B), PCs (outlined in Figure 1C) as well as anti-Qβ antibody titers (Figure 2). The donor derived secondary response was discriminated from the host's primary response using allotype specific detection antibodies for IgG1 and IgG2a in ELISA, as these are the main isotypes induced by Qβ immunization (46) (Figure 2). Donor derived antibodies after MBC transfer started to rise from day 4 after challenge, peaked around day 6 and then declined until day 20 (Figure 2A). In contrast, host antibody titers only started rising from day 6 and peaked at day 12. The peak titer of the host antibodies was lower than from the donor, indicating that MBC-derived secondary PCs dominated the early response. In addition, the relatively rapid decline of the donor-derived antibody titer is a clear indicator that the functional response of secondary PCs is unexpectedly short-lived.

Whether donor-derived MCBs could undergo a tertiary response was assessed next. To this end, recipient mice were challenged a second time with VLPs on day 61. Surprisingly, only the host-derived but not the donor-derived antibody response could be boosted, demonstrating that MBCs cannot participate twice in a humoral response after challenge with VLPs (Figure 2A). This suggests that essentially all MBCs generated against VLPs instantly differentiated to secondary PCs after re-stimulation without supplying a new MBC population. As expected, transferred naïve donor cells did not respond to the VLP challenge, as they also did not engage in the primary response (Figure 2B). Of note, host antibody levels were elevated after naïve B cell transfer compared to the host response in presence of MBCs (Figures 2A,B). This indicates that the presence of MBC derived secondary PCs suppresses the hosts humoral response after VLP challenge, confirming earlier observations (35).

In order to analyze the antibody avidity of the secondary antibody response, a modified ELISA was performed. For this purpose, low avidity antibodies were dissociated by treatment with 7 M urea. Only high avidity antibodies remain bound under these conditions (47, 48). Comparing the OD values of urea vs. PBS treated sera, an avidity index was calculated. The primary response antibodies of the host started to increase in avidity between day 6 and 9 after immunization (Figures 2C,D). The avidity increase proceeded until day 21. In marked contrast, avidity of antibodies derived from secondary PCs was high as of day 4 after challenge and did not further increase (Figure 2C). Thus, secondary PCs are not only superior in antibody production but also in antibody avidity.

### MBCs Do Not Extensively Proliferate Before Differentiating to Secondary PCs Upon Cognate Antigen Challenge

**Figure 3:**
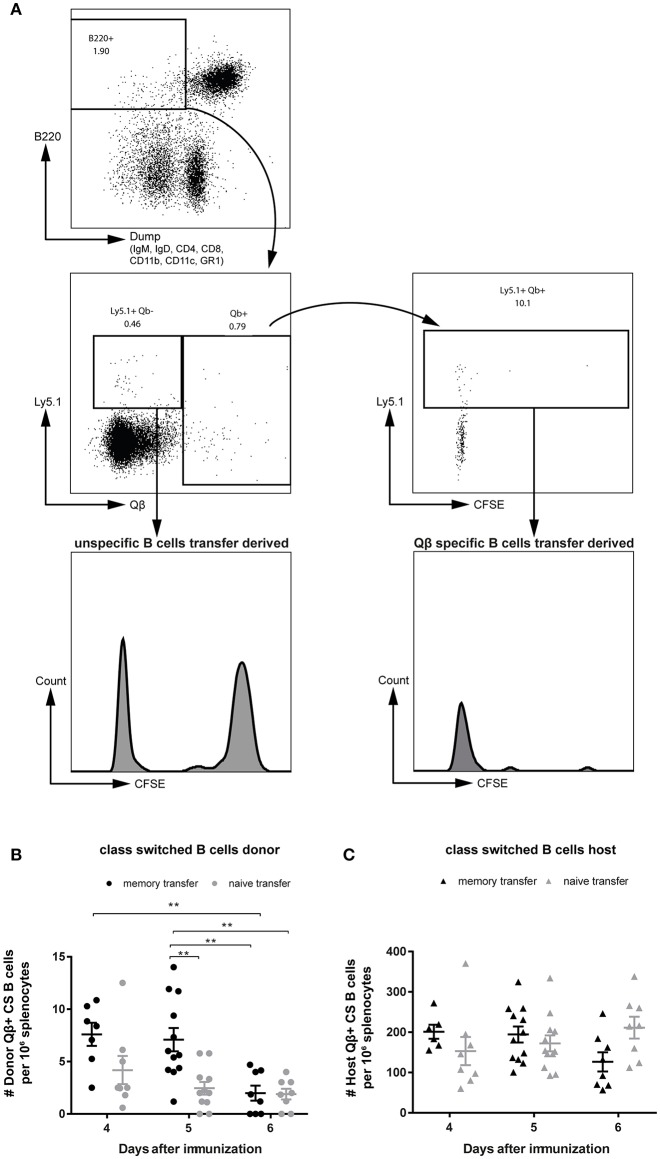
MBCs do not extensively proliferate before differentiating to secondary PCs upon cognate antigen challenge. PNA^−^, B220^+^ MACS purified cells from Qβ immune (8 weeks post immunization) or naïve Ly5.1 mice were labeled with CFSE and transferred into congenic hosts. Recipient mice were challenged 24 h later with 50 μg Qβ VLPs i.v. **(A)** Representative FCM plot to identify Qβ specific CS B cells in the spleen 5 days after challenge. B220^+^ cells negative for IgM, IgD, CD4, CD8, CD1b, CD11c, and GR1 were analyzed for their Qβ binding. CFSE dilution was examined to prove proliferation of donor derived cells (Ly5.1^+^). **(B,C)** Number of Qβ specific CS B cells on day 4, 5, and 6 after challenge identified by FCM as B220^+^, negative for IgM, IgD, CD4, CD8, CD11b, CD11c, GR1, and binding Qβ. Qβ specific donor **(B)** derived cells were distinguished from host derived cells **(C)** using the Ly5 congenic marker. Mean with SEM. *P* values were obtained using a one-way ANOVA followed by Tukey's multiple comparisons test. **p < 0.01. *n* = 4 mice per group. Data representative of at least 2 independent experiments.

To be able to study proliferation of MBCs before differentiation to PCs, purified B cell populations were labeled with CFSE prior to adoptive transfer. One day after transferring the MACS purified and CFSE labeled B cells, congenic recipient mice (Ly5.2) were challenged with 50 μg Qβ VLPs. Flow cytometric analysis of the Qβ specific CS B cells (Figure 1B) showed that all MBCs had proliferated as essentially no CFSE^+^ Ly5.1^+^ Qβ-specific cells could be observed (Figure 3A, right histogram). Nevertheless, there was a robust number of CFSE^+^, Ly5.1^+^, B220^+^ cells, which were not specific for Qβ, demonstrating survival of labeled cells upon adoptive transfer (Figure 3A, left histogram). The proliferation seen in this subset could be attributed to bystander proliferation or plasma blasts generated after proliferation and differentiation of MBCs, which had already downregulated surface BCR expression but are still B220^+^. Thus, essentially all MBCs proliferated but this proliferation was not extensive and of short duration, as few MBCs accumulated but rather rapidly differentiated into secondary PCs (see below).

### Transferred MBCs Are Detectable in the Specific B Cell Compartment Only at Early Time Points Upon VLP Challenge

In order to follow the MBC response upon transfer and challenge, the specific CS B cells were analyzed in the spleen by flow cytometry (FCM). VLP-specific CS B cells of donor (Figure 3B) and host (Figure 3C) origin were visualized as defined in Figure 1B and viable (Figures S2A,B). An increased number of Qβ specific donor derived cells was found, when MBCs were transferred compared to naïve B cell transfer on day 4 and 5 after VLP challenge (Figure 3B). This difference was more pronounced on day 5 but was already absent on day 6 post immunization. The host response in the CS B cell compartment was comparable between memory and naïve B cell transfer on day 4 and 5 after challenge (Figure 3C). However, the host B cell response seemed to be slightly impaired at day 6 when MBCs were present suggesting that MBCs suppress the response of the naïve host B cells (Figure 3C). This was consistent with the reduced host antibody titer in the presence of MBCs observed above (Figures 2A,B).

### Secondary PCs Are Rapidly Induced but Are Functionally Short-Lived

**Figure 4:**
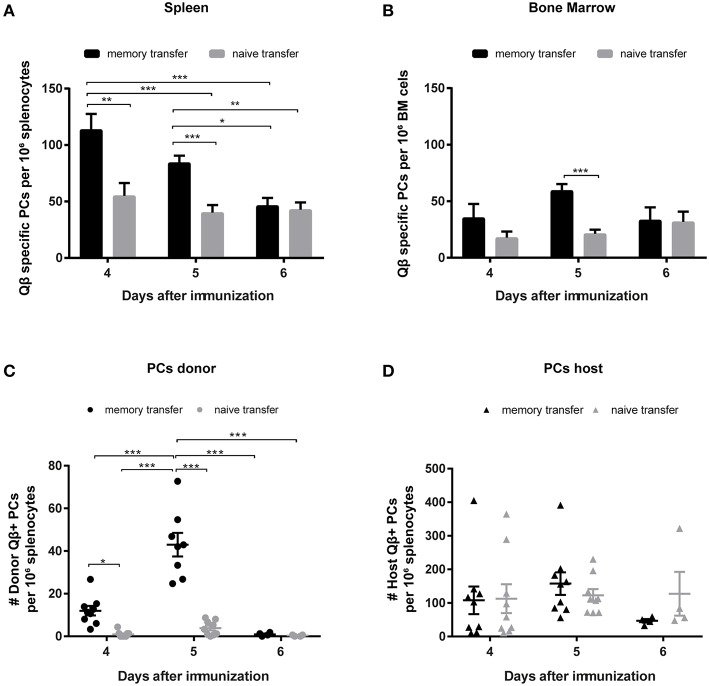
Transfer of memory B cells leads to an increased number of PCs, which are rapidly induced but short-lived. Congenic Ly5.1 mice were immunized with Qβ VLPs to generate MBCs. Eight weeks after the immunization PNA^−^, B220^+^ MACS purified cells from memory or naïve mice were transferred into congenic hosts. One day after the transfer, recipient mice were challenged with Qβ VLPs and the anti-Qβ PC response in spleen and BM was elucidated by ELISPOT and FCM. Number of Qβ specific PCs in spleen **(A)** and BM **(B)** on day 4, 5, and 6 after challenge determined by ELISPOT. FCM analysis of Qβ specific PCs within the B220^low^, IgM, IgD, CD4, CD8, CD11b, CD11c, and GR1 negative compartment, by intracellular Qβ binding after membrane permeabilisation. Qβ specific donor derived PCs **(C)** were distinguished from host derived PCs **(D)** using the Ly5 congenic marker. Mean with SEM. *P* values were obtained using a one-way ANOVA followed by Tukey's multiple comparisons test. *p < 0.05, **p < 0.01, ***p < 0.001. *n* = 4 mice per group. Data representative of at least 1–2 independent experiments.

To characterize the secondary PC population ELISPOT assays of spleen and BM were performed. As suggested by the antibody responses, secondary PCs occurred very promptly and reached high numbers 4 days after Ag challenge but the population rapidly contracted within the next 2 days (Figure 4A). Similar observations were made in the BM (Figure 4B). Besides ELISPOT analysis, PCs were enumerated by FCM, where the same pattern emerged. Secondary PCs occurred rapidly and peaked between days 4 and 5 but were largely absent by day 6 (Figure 4C). Thus, secondary PCs are induced within a few days but appear to be short-lived. As observed above for the CS B cells, the PC compartment of the host is similar on days 4 and 5, whereas it is slightly decreased on day 6 when MBCs were transferred (Figure 4D).

### Transfer Derived Secondary PCs Show Enhanced Capacity to Produce Antibodies in Spleen and BM

**Figure 5:**
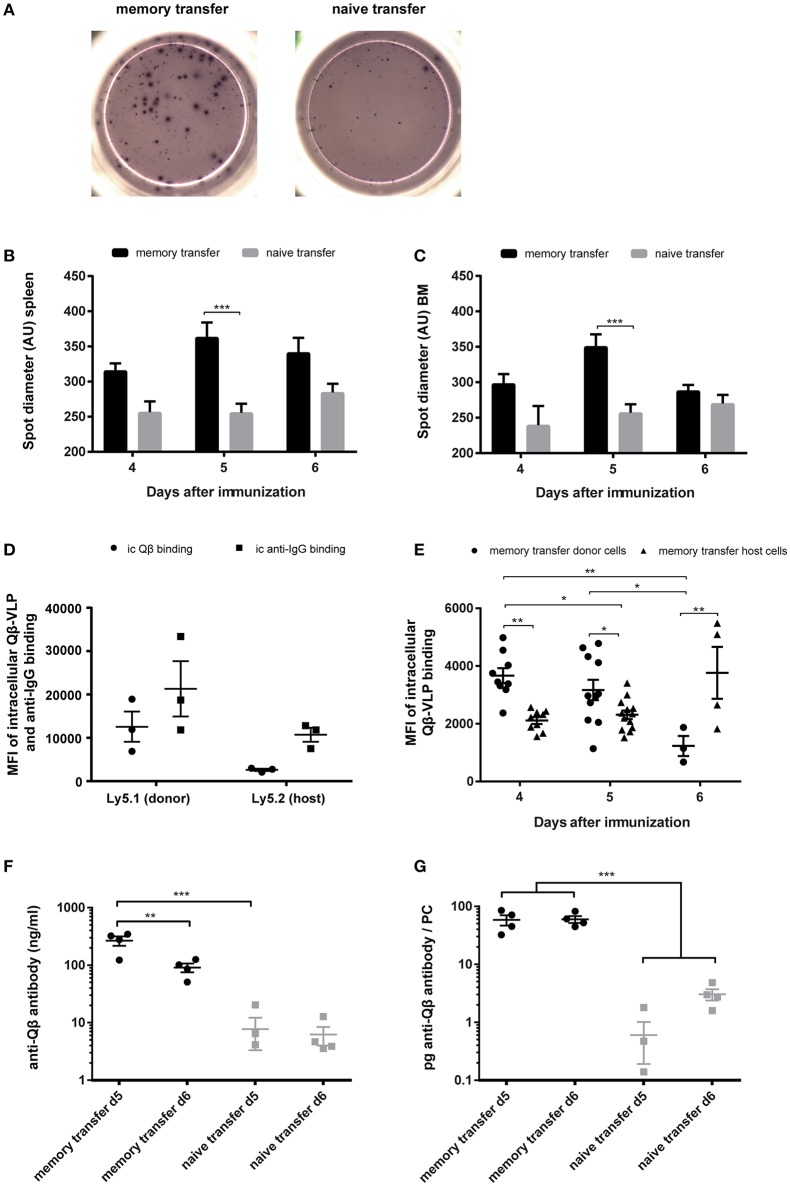
Transfer derived secondary PCs show enhanced capacity to produce antibodies in spleen and BM. PNA^−^, B220^+^ MACS purified cells from Qβ immune (8 weeks post immunization) or naïve Ly5.1 mice were transferred into congenic hosts. One day after the transfer, recipient mice were challenged with 50 μg Qβ VLPs i.v. Splenocytes and BM cells were analyzed by ELISPOT and FCM on day 4, 5, and 6 after challenge. **(A)** Representative images of anti-Qβ ELISPOTs on day 5 after challenge in the spleen. Quantification of spot diameter in spleen **(B)** and BM **(C)** on day 4, 5, and 6 after challenge. **(D)** Quantification of mean intracellular Qβ and anti-IgG binding of donor and host derived PCs on day 5 after memory transfer and challenge. **(E)** Quantification of mean intracellular Qβ-VLP binding of donor and host derived PCs after memory transfer. **(F,G)** Splenocytes were isolated 5 and 6 days after challenge and cultured for 3 days *in vitro*. Secreted antibodies in the cell supernatant were determined by ELISA. **(F)** Amount of anti-Qβ antibody secreted during the 72 h splenocyte cell culture. **(G)** Amount of specific antibodies produced per PC 5 and 6 days after memory or naïve cell transfer and challenge. Mean **(B,C,F,G)** or geometric mean **(D,E)** with SEM. *P* values were obtained using a one-way ANOVA followed by Tukey's multiple comparisons test. *p < 0.05, **p < 0.01, ***p < 0.001. *n* = 4 mice per group **(A–C,E–G)**, *n* = 3 mice per group **(D)**. Data representative of 1–2 independent experiments.

As previously described, one hallmark for secondary PCs is their enhanced capacity to produce antibodies after cognate antigen challenge (35). An indicator for enhanced antibody production during MBC responses was the spot size in ELISPOT assays, as it is correlating with the amount of antibodies produced by one PC. Representative images of ELISPOTs from splenocytes after memory or naïve B cell transfer and challenge with VLPs are shown (Figure 5A). Every spot on the plate represents one Qβ specific PC and the spot diameter correlates the amount of antibody that is produced by one PC. Spot diameters of specific PC populations in spleen and BM were analyzed 4–6 days after adoptive transfer of memory or naïve B cells that were challenged with VLPs. The spot diameter from spleen and BM was always greater when MBCs were transferred. The most significant difference however was observed on day 5 after Qβ VLP challenge, representing the peak of the secondary PC response (Figures 5B,C). This observation was confirmed by FCM on day 5 after MBC transfer and challenge, as the mean fluorescent intensity (MFI) of intracellular anti-IgG binding was increased in donor-derived compared to host PCs (Figure 5D). As the MFI of intracellular Qβ binding correlated with the amount of intracellular anti-IgG binding it can serve as a surrogate for the amount of antibody present inside PCs (Figure 5D). The MFI of intracellular Qβ binding was significantly increased 4 and 5 days after challenge with Qβ when MBCs were transferred (Figure 5E). Taken together the results of the spot size and intracellular staining of spleen and BM-derived PCs indicated that secondary PCs produced increased amounts of antibodies.

Both spot size and intracellular staining with Qβ-VLPs may not linearly correlate with antibody production. To estimate the amount of antibodies produced by secondary vs. primary PCs more directly, splenocytes were collected and cultured from mice 5 and 6 days after adoptive transfer and VLP challenge. Whole splenocytes were seeded into 24 well-plates for 72 h and frequencies of specific PCs were quantified by FCM at the beginning of the culture. The amount of anti-Qβ antibody in cell culture supernatants of splenocytes harvested 5 and 6 days after challenge was ~30 fold increased when MBCs were transferred, again demonstrating that secondary PCs produced elevated antibody levels (Figure 5F). The total amount of anti-Qβ antibody decreased from day 5 to day 6 after memory transfer (Figure 5F) further demonstrating their short lived nature. Nevertheless, the amount of specific antibody per PC stayed the same (Figure 5G). After naïve transfer, on the contrary, the amount of antibody per PC increased over time, as the primary response evolved (Figure 5G). This massively increased protein production by secondary PCs illustrates the stress these PCs may be exposed to, probably resulting in the short live span.

## Discussion

Long-lived PCs are crucial for sustained immune protection through secretion of specific antibodies (24). However, PCs do not always become long-lived during infection or vaccination because most of the PCs die early during the immune response. In fact, during primary immunogenicity studies using VLPs, we observed that the PC population in spleen emerged on day 4, peaked at day 7 and subsequently declined rapidly, followed by a phase of more stable PC frequencies (35). Hence, most PCs formed initially against Qβ are short-lived (41). This short lifespan may be a result of the irrevocable cell cycle arrest which PCs usually enter and therefore cannot maintain a cellular pool by means of proliferation. In contrast, the state of irreversible cell cycle quiescence must be controlled by mechanisms to enable long-term PC survival. Moreover, the ephemerality of the early PCs could also be due to ER stress caused by the massive antibody production. Cell intrinsic constraints like unfolded protein response (UPR) and autophagy can rescue PCs from cell death but the cells additionally require sufficient nutrients, external survival signals and a survival niche (30, 49–52). Long-lived PCs are found in both the spleen and BM. However, the numbers of niches is finite, thus restricting the number of PCs with access to them (1). In fact, most VLP specific PCs reaching the BM do not survive as the number of PCs rapidly declines initially also in the BM (45). The constant competition for space and survival signals of PCs within the BM may provide an opportunity to manipulate PC survival for long-term antibody production upon vaccination as well as for therapies of malignant PC diseases (53). Moreover, CD28 has been shown to be expressed by human and mouse PCs (54, 55). Engagement of CD28 with CD80/CD86 derived from cellular partners in the PC niche was demonstrated to be important for BM long-lived PC survival, half-life and sustained antibody responses. Downstream signaling of CD28 induces BLIMP1 upregulation and is therefore involved in regulating PC differentiation and maintenance (55, 56). Furthermore, CD28 was shown to regulate glycolysis in long-lived PCs providing glycolytic end products for oxidative energy production and biosynthesis (57). Additionally CD28 regulated mitochondrial metabolism and respiration which favored survival of long-lived PCs (58, 59). In contrast to long-lived PCs, CD28 exhibited a higher activation threshold in short-lived PCs and therefore had no positive impact on their survival (55). Together with limited access to CD80/CD86 molecules derived from BM PC niche cells, the increased threshold of CD28 activation could be reasons for the short-lived nature of the secondary PCs. We are currently assessing a potential role of CD28 in the lifespan of primary and secondary PCs.

A population of MBCs is maintained after the decline of immune responses and may be activated upon re-infection to rapidly differentiate into PCs, which secrete antibodies. However, it has also been reported that MBCs can re-enter GCs and interact with T follicular helper cells shaping the immune response and generating a new pool of MBCs. In analogy to memory T cells, these two different effector functions of MBCs define two distinct cellular compartments: the effector MBCs differentiating into PCs for rapid antibody production and the central MBCs playing a role in re-initiating the GC response and maintaining the MBC pool (60). The secondary PCs described herein are derived from effector MBCs that were generated by a single round of immunization using Qβ-VLP. B cell intrinsic toll-like receptor (TLR) 7 stimulation was shown to be essential for MBC generation that were capable of differentiating to secondary PCs (61). Intriguingly, VLP specific MBCs only responded a single time to Ag re-stimulation, namely by terminal differentiation into short-lived PCs. These secondary PCs were B220^−^ and no longer carried their Ig on the surface and therefore could not be further stimulated with the Ag. Baptista et al. studied PC differentiation in response to innate stimuli in the absence of antigen and observed that TLR9 signaling by CpG failed to differentiate follicular B cells into PCs whereas TLR4 stimulation by lipopolysaccharide (LPS) induced antibody production, PC surface markers such as CD138 and canonical transcription factors like IRF4, BLIMP1 or XBP1 (62). Furthermore, ligation of BCR and TLR7 was shown to drive PC differentiation (63, 64). Therefore, the downregulation of BCR and B220 expression and increased antibody production seen in response to MBC re-stimulation with VLPs containing bacterial RNA are clear signs of PC differentiation. Nevertheless, we never found a homogenous CD138^+^ cell population using VLPs for vaccination. Consequently, further work needs to be done to determine the expression of classical PC transcription factors and surface markers to characterize the phenotype of secondary PCs in more detail.

The avidity of the antibodies secreted by secondary PCs was very high at early time points after immunization, a level which antibodies generated during a primary response only reached by day 20 upon VLP immunization. This finding is consistent with the notion that secondary PCs derive from MBCs which have undergone avidity maturation in a GC reaction (45). Thus, secondary PCs provide the host with a rapid wave of high-avidity antibodies. The great amount of VLP-specific IgG, which is present early during the recall response, is most likely responsible for the suppression of the host response. Link et al. demonstrated that VLPs complexed to specific IgGs were taken up by macrophages in the subcapsular sinus and did not efficiently reach B cell follicles and follicular dendritic cells, leading to antigen deprival for naïve B cell activation (65).

Surprisingly, secondary PCs did not have a long functional lifespan, neither in spleen nor in BM. In fact, most of them disappeared from lymphoid organs within 6 days after Ag re-stimulation. The dominant pool of long-lived PCs induced in the presence of MBC was derived from primary B cells. The early death of secondary PCs is probably a consequence of the enhanced antibody production, which increases cellular stress levels. Access to niches seems less important, as numbers of primary and secondary PCs are similar at day 6 after challenge, and yet only primary PCs become long-lived. The increased antibody production in secondary PCs more likely accounts for the short lifespan due to increased ER stress as well as accelerated demand for nutrients. The fact that secondary PCs produce at least 30 times more antibody than primary PCs underscores this point. It has been shown that PCs are able to adapt their metabolism according to the changing environment, but secondary PCs may be induced too quickly to produce very large amounts of antibodies, to be able to adapt their metabolism sufficiently. In addition, they may not live long enough to actually find a niche allowing their long-term survival. In this respect, secondary PCs behave more like innate cells, which usually respond very rapidly but are short-lived as well. In terms of surface marker and transcription factor expression, secondary PCs most likely do not differ extensively from other PC populations. However, there are substantial functional differences.

The fact that during viral infection all MBCs differentiate into functionally short-lived secondary PCs has interesting biological implications as it keeps the antibody repertoire flexible and adaptable to the changing world of pathogens, as e.g., influenza viruses. Secondary PCs produce an early wave of high avidity antibodies specific for the strain of pathogen previously encountered. Under this early protective antibody umbrella, naïve B cells are activated, initiate a novel GC reaction and generate high avidity PCs and MBCs for the current version of the pathogen. This mechanism ensures that the antibody repertoire is not frozen to the specificity for a single version of a pathogen but remains adaptable to their evolution. In this respect, the here presented mechanism ensures that original antigenic sin does not limit the dynamics and broadness of the antibody repertoire too extensively (25, 66, 67).

In summary, we demonstrate here that upon challenge with viral particles MBCs differentiate rapidly into secondary PCs, providing the host with an early wave of high avidity antibodies. Thus, induction of effector MBCs, which can provide rapid and effective protection by differentiating into secondary PCs, may be a promising alternative that should be considered in vaccine development.

## Ethics Statement

This study was carried out in accordance with the recommendations of the Bundesamt für Lebensmittelsicherheit und Veterinärwesen (BLV) and the guidelines of the Cantonal Veterinary Office Bern, Switzerland. The protocol was approved by the Cantonal Veterinary Office Bern, Switzerland.

## Author Contributions

CK and EK performed all experiments. CK, FT, and MB designed all the experiments and wrote the manuscript. MV interpreted results and contributed to the scientific discussion. All authors read and commented on the manuscript.

### Conflict of Interest Statement

The authors declare that the research was conducted in the absence of any commercial or financial relationships that could be construed as a potential conflict of interest.
