# Supplementary material for: Virus-Specific Secondary Plasma Cells Produce Elevated Levels of High-Avidity Antibodies but Are Functionally Short Lived
Source: Front Immunol. 2019 Aug 6;10:1831. doi: 10.3389/fimmu.2019.01831 (PMC6691049; doi:10.3389/fimmu.2019.01831)
Supplement: Supplementary file 1 [file Data_Sheet_1.PDF]

Supplementary materials

Figure S1

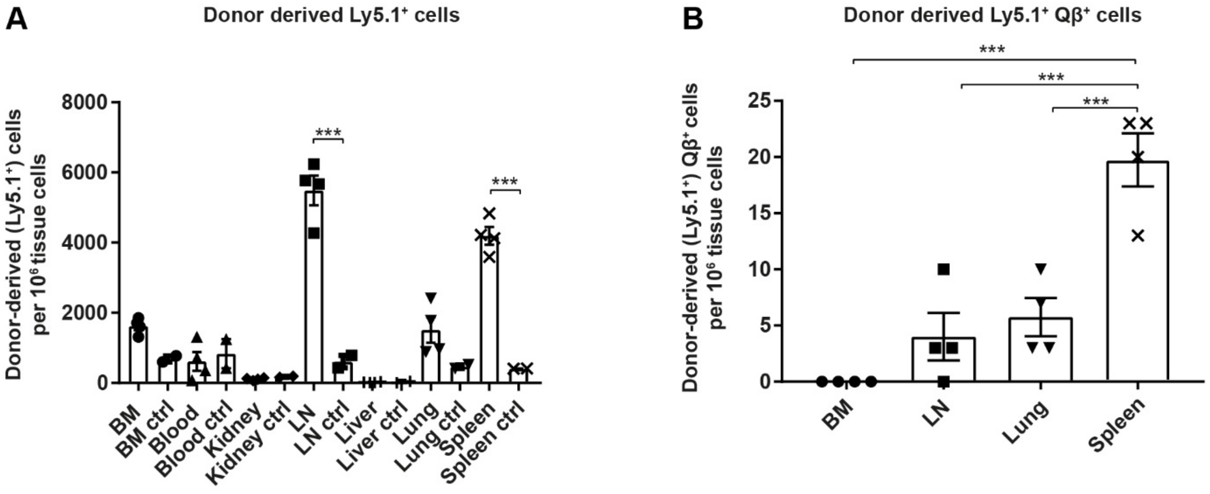

Figure S1: Tissue distribution of Ly5.1^{+} cells after transfer. Unpurified cells from Qβ immune Ly5.1 mice were transferred to allotypic (Ly5.2) hosts and their tissue distribution was analysed 4 days after the transfer. (**A**) The number of donor-derived (Ly5.1^{+}) cells in the BM, blood, kidney, lymph nodes (LN), liver, lung and spleen was quantified by FCM. Naïve Ly5.2 mice served as controls (ctrl). (**B**) The number of Qβ VLP-specific donor-derived cells was quantified in the BM. LN, lung and spleen at day 4 after the transfer. Mean with SEM. P values were obtained using a one-way ANOVA followed by Sidak's multiple comparisons test comparing all groups to the respective control. *** $p < 0.001$ n = 4 mice per group and 2 naïve controls. Data representative of 1 experiment.

Figure S2

A Viability of Qβ specific CS B cells in spleen, d6 p. i.

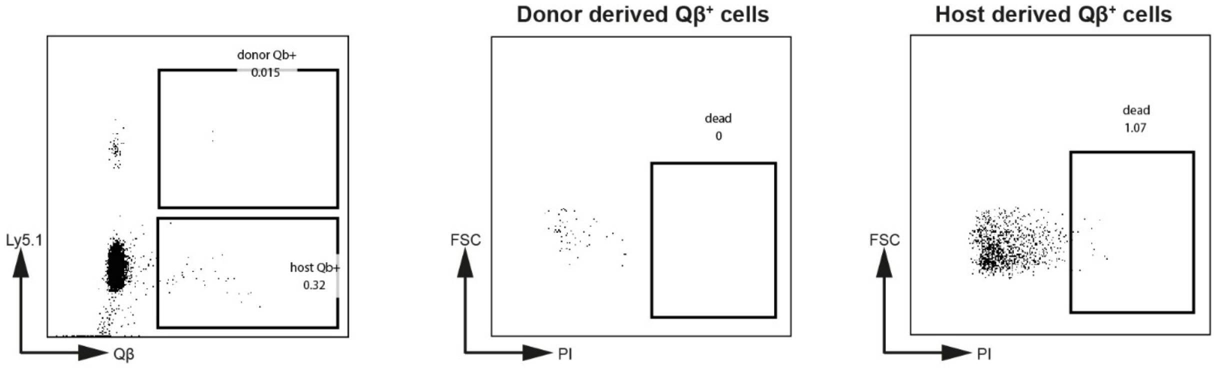

B Viability of Qβ specific PCs in spleen, d6 p. i.

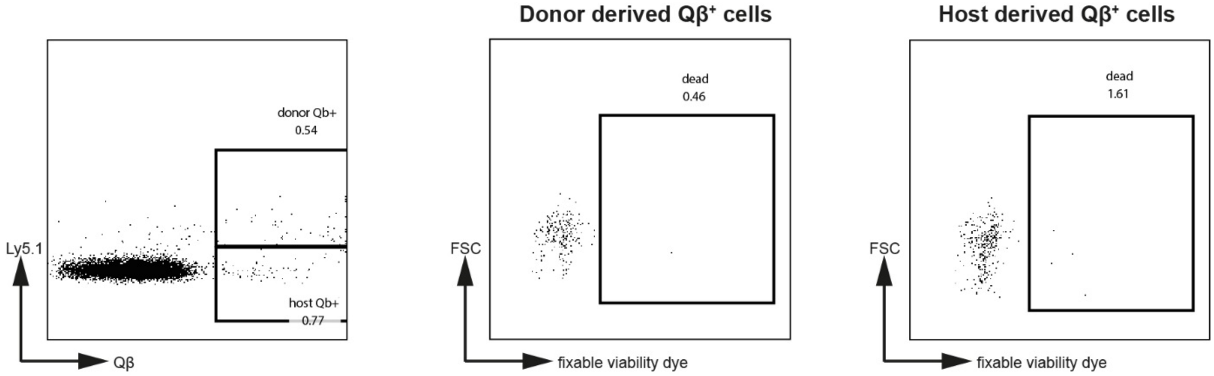

Figure S2: Viability of Qβ-specific CS B cells and PCs. Congenic mice (Ly5.1) were immunized with 50 µg Qβ VLPs i.v.. Eight weeks after immunization spleens of immunized and naïve mice were isolated and PNA^- $B220^+$ MACS purified cells were transferred into host mice (Ly5.2). Recipient mice were immunized with 50 µg Qβ VLPs i.v. one day after the transfer. The viability of $Q\beta^+$ CS B cells and PCs in the spleen was assessed 6 days after challenge. **(A)** Representative FCM plots of $B220^+$ cells not expressing IgM, IgD, CD4, CD8, CD11b, CD11c or GR1 and binding labelled Qβ VLPs. The congenic Ly5 marker was used to discriminate transfer from host derived CS B cells. Dead cells amongst donor and host derived Qβ-specific CS B cells were identified using propidium iodide (PI). **(B)** Representative FCM plots of $B220^{low}$ cells not expressing IgM, IgD, CD4, CD8, CD11b, CD11c or GR1 and binding labelled Qβ VLPs intracellularly after permeabilisation. The congenic Ly5 marker was used to discriminate transfer from host derived PCs cells. Dead cells amongst donor and host derived Qβ-specific PCs were stained using a fixable viability dye.
